# Association between inpatient aspirin exposure and 28-day mortality in critically ill patients with sepsis-induced coagulopathy: A cohort study

**DOI:** 10.1097/MD.0000000000051494

**Published:** 2026-11-06

**Authors:** Jiantao Zheng, Yinying Wang, Chanjuan Zhou, Siming Lin

**Affiliations:** a Department of Emergency Medicine, Quanzhou First Hospital Affiliated to Fujian Medical University, Quanzhou, Fujian, China; b Department of Infectious Disease, Beijing Luhe Hospital, Capital Medical University, Beijing, China; c Department of General practice, Quanzhou First Hospital Affiliated to Fujian Medical University, Quanzhou, China; d Department of Emergency, The First Affiliated Hospital, Fujian Medical University, Fuzhou, Fujian, China; e Department of Emergency, National Regional Medical Center, Binhai Campus of the First Affiliated Hospital, Fujian Medical University, Fuzhou, Fujian, China.

**Keywords:** aspirin, coagulation disorders, critical care, mortality, sepsis

## Abstract

This study aimed to evaluate the association between inpatient aspirin exposure and 28-day mortality in critically ill patients with sepsis-induced coagulopathy (SIC). We conducted a retrospective cohort study extracting data from the MIMIC-IV v3.1 database (2008–2022). Adult critically ill patients with SIC in the intensive care unit (ICU) were included. The primary outcome was 28-day mortality. Secondary outcomes encompassed mortality (e.g., in-hospital, ICU, 60-day, 90-day, and 1-year) and duration of ICU-free, ventilation-free, or vasopressor-free days within 28 days of admission to the ICU. Multivariable Cox regression, propensity score matching (PSM), inverse probability of treatment weighting (IPTW), overlap weighting (OW), pairwise algorithmic weighting (PA), and doubly robust estimation were employed to control confounders. Robustness of findings was assessed through subgroup and sensitivity analyses. Among 9095 critically ill patients with SIC, 3207 (35.3%) received aspirin during ICU hospitalization. After 1:1 propensity score matching (2069 pairs), inpatient aspirin exposure was independently associated with significantly lower 28-day mortality (hazard ratio [HR] 0.74; 95% confidence interval [CI]: 0.65–0.85; *P* < .001). This association remained consistent across multiple analytical methods including IPTW (HR 0.76; 95% CI: 0.70–0.84; *P* < .001), OW (HR 0.72; 95% CI: 0.61–0.84; *P* < .001), PA (HR 0.73; 95% CI: 0.61–0.84; *P* < .001), and doubly robust estimation (HR 0.76; 95% CI: 0.61–0.94; *P* < .001). Inpatient aspirin exposure was also associated with more favorable secondary clinical outcomes (all *P* < .001). In critically ill patients with SIC, inpatient aspirin exposure was independently associated with lower 28-day mortality and more favorable clinical outcomes. These findings should be interpreted as an association rather than evidence of a causal therapeutic effect. Prospective randomized studies are warranted to validate these findings.

## 1. Introduction

Sepsis-induced coagulopathy (SIC) involves dysregulated coagulation-fibrinolysis pathways that drive high mortality in critically ill patients in the intensive care unit (ICU), the incidence of which reaches 66% in septic shock cohorts.^[1,2]^ In the pathophysiological mechanism of SIC, the intricate interactions of systemic inflammation, endothelial injury, and coagulation cascade activation promote organ dysfunction and elevate mortality risk.^[3]^ Despite diagnostic and therapeutic advances, SIC prognosis remains poor, underscoring the need for novel interventions. Recent literature on therapeutic strategies for SIC pointed out that anticoagulants and unfractionated heparin (UFH) confer survival benefits for critically ill patients with SIC.^[4]^ Coagulation modulators (e.g., recombinant thrombomodulin) demonstrated efficacy against sepsis-induced disseminated intravascular coagulation (DIC).^[5]^

Aspirin, a cornerstone antiplatelet agent in cardiovascular secondary prevention, demonstrates emerging multifaceted therapeutic potential in sepsis through platelet inhibition, anti-inflammatory activity, endothelial protection, and microcirculatory improvements. Retrospective Medical Information Mart for Intensive Care IV (MIMIC-IV) analyses demonstrate improved survival with aspirin in sepsis^[6]^ and sepsis-associated conditions (e.g., acute kidney injury [SA-AKI],^[7]^ myocardial injury (SA-MI),^[8]^ encephalopathy (SAE),^[9]^ acute respiratory failure (SA-ARF)^[10]^ and liver injury (SA-LI)^[11]^). However, large-scale studies (e.g., retrospective analyses or randomized controlled trials) specifically evaluating aspirin’s impact on mortality endpoints in SIC remain scarce. Consequently, the impact of aspirin on both short-term and long-term mortality among SIC patients remains an important and unresolved clinical question.

Leveraging the MIMIC-IV database (v3.1), this retrospective cohort study evaluated the association between inpatient aspirin exposure and clinical outcomes among critically ill patients with SIC, with particular emphasis on 28-day mortality. Our objective was to provide observational evidence to inform future prospective investigations rather than establish a causal therapeutic effect.

## 2. Materials and methods

### 2.1. Study design

The data for this retrospective cohort study were extracted from version 3.1 (v3.1) of the MIMIC-IV database (https://physionet.org/content/mimiciv/3.1/), a publicly available repository. This database contains anonymous medical records for 94,458 adult patients in the ICU treated at Beth Israel Deaconess Medical Center (BIDMC) from 2008 to 2022.^[12]^ The study protocol was approved in advance by the Institutional Review Board of Beth Israel Deaconess Medical Center. The first author gained access permission (certification number: 56456740) after completing required certification and signing a data use agreement. Additionally, the ethics committee waived the requirement for written informed consent because all data in the database are fully anonymized. The investigation complies with the Strengthening the Reporting of Observational Studies in Epidemiology (STROBE)^[13]^ guidelines for reporting observational studies in epidemiology.

### 2.2. Study setting and population

Patients with SIC admitted to the ICU within the first 24 hours between 2008 and 2022 were included in this study. The diagnosis of sepsis was based on the criteria established by the Third International Consensus Definitions (Sepsis 3.0) for Sepsis and Septic Shock, characterized by confirmed or suspected infection along with a Sequential Organ Failure Assessment (SOFA) score of 2 or higher.^[14]^ Following this, the diagnosis of SIC required only 2 laboratory parameters: platelet (PLT) counts and international normalized ratio (INR) according to the criteria and its scoring system constructed in 2017^[15]^ (Table S1, Supplemental Digital Content 1). The exclusion criteria were as follows: not first ICU admissions (n = 13560); missing PLT counts (n = 78) or INR (n = 1584) on the first day of ICU admission; pregnant women (n = 33); ICU stays shorter than 48 hours (n = 5071).

### 2.3. Study protocol and measures

Data extraction from MIMIC-IV v3.1 was performed using PostgreSQL (version 13.6.1) and Navicat Premium (version 17.2) via SQL queries.

Aspirin exposure was defined as at least 1 aspirin prescription during the ICU hospitalization period in the MIMIC-IV database medication records, including both the continuation of pre-admission aspirin use in the ICU and newly initiated aspirin after admission. Identification was performed by searching for the drug compound “aspirin” and excluding entries such as “Aspirin Desensitization.” Routes of administration included oral and nasogastric tube administration. In addition to aspirin exposure, the extracted variables were shown in Table 1. Baseline data were collected within the first 24 hours after ICU admission. Potential confounding factors (Table 2) were selected based on univariable analysis results (*P *< .1; Table S2, Supplemental Digital Content 2), existing literature, and clinical judgment. Multicollinearity was then assessed using variance inflation factors, with values <5 indicating no multicollinearity. Variables with missing data rates below 30% (Table S3, Supplemental Digital Content 3) were addressed using an iterative multivariate single-imputation approach.^[16]^

**Table 1:** Baseline characteristics and outcomes.

Patient characteristic	Total (n = 9095)	Non-aspirins (n = 5888)	aspirins (n = 3207)	*P*
Demographics				
Age (yr)	66.6 ± 16.1	64.2 ± 17.0	71.0 ± 13.2	<.001
Gender, male, n(%)	5568 (61.2)	3497 (59.4)	2071 (64.6)	<.001
Race, non-White, n(%)	3201 (35.2)	2144 (36.4)	1057 (33)	<.001
BMI (kg/m^2^)	29.3 ± 7.0	29.2 ± 7.3	29.4 ± 6.6	.144
Baseline vital signs				
Heart rate (beats/min)	89.1 ± 17.0	91.3 ± 17.7	85.0 ± 14.8	<.001
Mean blood pressure (mm Hg)	75.6 ± 9.5	76.0 ± 10.0	74.9 ± 8.5	<.001
RR (breaths/min)	20.1 ± 4.3	20.5 ± 4.5	19.3 ± 3.8	<.001
Temperature (°C)	36.9 ± 0.6	36.9 ± 0.6	36.8 ± 0.6	<.001
SpO_2_ (%)	97.1 ± 2.2	96.9 ± 2.3	97.3 ± 2.0	<.001
Comorbidity disease, n(%)				
AKI	8281 (91.1)	5291 (89.9)	2990 (93.2)	<.001
CHF	3174 (34.9)	1664 (28.3)	1510 (47.1)	<.001
CVD	1275 (14.0)	729 (12.4)	546 (17)	<.001
COPD	2256 (24.8)	1395 (23.7)	861 (26.8)	<.001
CLD	2348 (25.8)	1899 (32.3)	449 (14)	<.001
PVD	1365 (15.0)	599 (10.2)	766 (23.9)	<.001
Diabetes	2729 (30.0)	1598 (27.1)	1131 (35.3)	<.001
Malignancy	1434 (15.8)	1165 (19.8)	269 (8.4)	<.001
MI	1728 (19.0)	643 (10.9)	1085 (33.8)	<.001
Peptic ulcer disease	349 (3.8)	268 (4.6)	81 (2.5)	<.001
Rheumatic disease	333 (3.7)	213 (3.6)	120 (3.7)	.763
Laboratory measurements				
WBC (×10^9^/L)	14.6 (10.0, 20.1)	14.2 (9.5, 20.2)	15.2 (11.0, 19.9)	<.001
Hemoglobin (g/dL)	9.2 ± 2.2	9.3 ± 2.2	9.2 ± 2.0	.044
PLT (×10^9^/L)	116.0 (77.0, 166.0)	112.0 (69.0, 169.0)	122.0 (90.0, 164.0)	<.001
Sodium (mmol/L)	136.3 ± 5.8	136.1 ± 6.2	136.7 ± 4.8	<.001
Potassium(mmol/L)	4.7 ± 0.9	4.7 ± 0.9	4.8 ± 0.9	.009
Calcium(mg/dL)	7.8 ± 0.9	7.8 ± 1.0	8.0 ± 0.9	<.001
Creatinine (mg/dL)	1.3 (0.9, 2.2)	1.4 (0.9, 2.3)	1.2 (0.9, 2.0)	<.001
BUN (mg/dL)	27.0 (17.0, 45.0)	28.0 (18.0, 47.0)	24.0 (17.0, 39.0)	<.001
Glucose (mg/dL)	133.8 (116.0, 164.0)	133.4 (112.1, 166.1)	134.2 (120.9, 161.0)	<.001
Lactate (mmol/L)	2.9 (1.8, 4.8)	2.8 (1.7, 4.8)	3.0 (2.0, 4.9)	<.001
AG (mmol/L)	17.3 ± 5.7	17.9 ± 5.8	16.3 ± 5.3	<.001
Bicarbonate (mmol/L)	19.9 ± 5.0	19.6 ± 5.3	20.4 ± 4.4	<.001
INR	1.6 (1.5, 2.1)	1.7 (1.5, 2.3)	1.6 (1.4, 1.9)	<.001
PT (s)	18.0 (15.8, 23.1)	18.4 (15.9, 24.2)	17.4 (15.8, 21.1)	<.001
PTT (s)	51.7 ± 33.5	49.0 ± 31.0	56.6 ± 37.2	<.001
Disease assessment indicators				
APS III score	65.7 ± 28.4	68.9 ± 28.6	59.7 ± 26.9	<.001
CCI	6.2 ± 2.9	6.1 ± 3.0	6.6 ± 2.6	<.001
OASIS score	37.9 ± 9.3	38.3 ± 9.3	37.2 ± 9.2	<.001
SAPS II score	44.4 ± 14.5	44.7 ± 14.9	43.8 ± 13.8	.007
SOFA score	7.5 ± 3.5	7.7 ± 3.7	7.2 ± 3.1	<.001
SIC score n (%)				<.001
4	4333 (47.6)	2737 (46.5)	1596 (49.8)	
5	2460 (27.0)	1513 (25.7)	947 (29.5)	
6	2302 (25.3)	1638 (27.8)	664 (20.7)	
Intervention measures, n (%)				
Heparin	6994 (76.9)	4635 (78.7)	2359 (73.6)	<.001
Statins	2894 (31.8)	889 (15.1)	2005 (62.5)	<.001
Vasopressor	5594 (61.5)	3337 (56.7)	2257 (70.4)	<.001
MV	6031 (66.3)	3664 (62.2)	2367 (73.8)	<.001
CRRT	1411 (15.5)	933 (15.8)	478 (14.9)	.236
Outcomes				
Los-ICU (days)	4.7 (3.0, 8.7)	4.7 (3.0, 8.6)	4.8 (3.0, 8.8)	.584
In-hospital mortality, n (%)	2976 (21.0)	2352 (25.4)	624 (12.6)	<.001
ICU mortality, n (%)	2237 (15.8)	1776 (19.2)	461 (9.3)	<.001
28 d mortality, n (%)	2323 (25.5)	1767 (30)	556 (17.3)	<.001
60 d mortality, n (%)	2787 (30.6)	2089 (35.5)	698 (21.8)	<.001
90 d mortality, n (%)	3007 (33.1)	2237 (38)	770 (24)	<.001
1 yr mortality, n (%)	3702 (40.7)	2699 (45.8)	1003 (31.3)	<.001

Data are presented as mean ± SD, medians [IQR], or frequency (percentage).

AG = anion gap, AKI = Acute Kidney Injury, APS III = Acute Physiology Score III, BMI = body mass index, BUN = blood urea nitrogen, CCI = Charlson comorbidity index, CHF = congestive heart failure, CLD = chronic liver disease, COPD = chronic obstructive pulmonary disease, CRRT = continuous renal replacement therapy, CVD = cerebrovascular disease, ICU = intensive care unit, INR = international normalized ratio, IQR = interquartile range, MI = myocardial infarction, MV = Mechanical ventilation, OASIS = Oxford acute severity of illness score, PLT = platelet, PT = prothrombin time, PTT = partial thromboplastin time, PVD = peripheral vascular disease, RR = respiratory rate, SAPS II = simplified acute physiology score II, SD = standard deviation, SIC = sepsis-induced coagulopathy, SOFA = sequential organ failure assessment, SpO_2_ = oxygen saturation, WBC = white blood cell count.

**Table 2:** Imbalance of patient characteristics before and after propensity score matching in the assessment of 28-day mortality.

Variables	Unmatched			Matched		
	Non-aspirins	Aspirins	SMD	Non-aspirin	Aspirins	SMD
n	5888	3207		2069	2069	
Age (yr)	64.18 (16.98)	71.00 (13.19)	0.448	70.24 (14.34)	69.43 (14.32)	0.057
Male (%)	3497 (59.4)	2071 (64.6)	0.107	1287 (62.2)	1300 (62.8)	0.013
Non-White (%)	2144 (36.4)	1057 (33.0)	0.073	709 (34.3)	700 (33.8)	0.009
BMI (kg/m^2^)	28.90 (7.10)	29.29 (6.68)	0.057	29.37 (7.04)	29.20 (6.90)	0.024
RR (bpm)	20.46 (4.46)	19.32 (3.81)	0.276	19.66 (3.97)	19.70 (4.00)	0.012
AG (mmol/L)	17.86 (5.81)	16.25 (5.30)	0.288	16.92 (5.17)	16.86 (5.51)	0.012
BUN (mg/dL)	37.14 (28.15)	32.03 (23.49)	0.197	35.16 (25.56)	33.99 (25.35)	0.046
Lactate (mmol/L)	3.68 (3.26)	3.72 (2.63)	0.015	3.61 (2.96)	3.71 (2.66)	0.036
MI (%)	643 (10.9)	1085 (33.8)	0.572	487 (23.5)	472 (22.8)	0.017
Liver disease (%)	1899 (32.3)	449 (14.0)	0.443	376 (18.2)	387 (18.7)	0.014
Malignant neoplasms (%)	1165 (19.8)	269 (8.4)	0.332	253 (12.2)	235 (11.4)	0.027
AKI (%)	5291 (89.9)	2990 (93.2)	0.121	1907 (92.2)	1903 (92.0)	0.007
APS III score	68.89 (28.61)	59.71 (26.89)	0.33	62.52 (26.63)	62.44 (27.46)	0.003
SIC score (%)			0.169			0.02
4	2737 (46.5)	1596 (49.8)		1053 (50.9)	1047 (50.6)	
5	1513 (25.7)	947 (29.5)		568 (27.5)	585 (28.3)	
6	1638 (27.8)	664 (20.7)		448 (21.7)	437 (21.1)	
CCI	6.06 (3.03)	6.57 (2.62)	0.178	6.56 (2.76)	6.41 (2.74)	0.058
Statins (%)	889 (15.1)	2005 (62.5)	1.114	861 (41.6)	879 (42.5)	0.018
Heparin (%)	4635 (78.7)	2359 (73.6)	0.121	1581 (76.4)	1569 (75.8)	0.014
CRRT (%)	933 (15.8)	478 (14.9)	0.026	308 (14.9)	308 (14.9)	<0.001
Vasopressor (%)	3337 (56.7)	2257 (70.4)	0.288	1338 (64.7)	1337 (64.6)	0.001
MV (%)	3664 (62.2)	2367 (73.8)	0.25	1387 (67.0)	1409 (68.1)	0.023
Los-ICU	7.14 (7.24)	7.68 (8.41)	0.069	7.31 (8.23)	7.32 (7.68)	0.001

An absolute MSD of <10% was considered to support the assumption of a balance between the groups. SMD, standardized mean differences. Data are presented as mean ± SD, or as frequency (percentage).

AG = anion gap, AKI = acute kidney injury, APS III = Acute Physiology Score III, BMI = body mass index, BUN = blood urea nitrogen, CCI = Charlson Comorbidity Index, CRRT = continuous renal replacement therapy, IQR = interquartile range, LOS-ICU = length of stay in intensive care unit, MI = myocardial infarction, MV = mechanical ventilation, RR = respiratory rate, SD = standard deviation, SIC = sepsis-induced coagulopathy, SMD = standardized mean difference.

The primary outcome was 28-day mortality. Secondary outcomes encompassed mortality (e.g., in-hospital, ICU, 60-day, 90-day, and 1-year), and duration of ICU-free, ventilation-free, or vasopressor-free days within 28 days of admission to ICU.

### 2.4. Data analyses

Continuous variables were assessed for normality. Normally distributed data are reported as mean with standard deviation (mean ± SD), while non-normally distributed variables are expressed as median with interquartile range (IQR). Between-group comparisons employed the Student’s *t* test or Wilcoxon rank-sum test, as appropriate. Categorical variables are presented as frequency (percentage), with comparisons performed using Pearson’s χ^2^ or Fisher’s exact tests.

Multivariable Cox proportional hazards regression was performed to evaluate the independent association between aspirin use and 28-day mortality, estimating hazard ratios (HRs) with 95% confidence intervals (CIs). Survival curves were generated using Kaplan–Meier analysis and compared via the log-rank test.

Propensity scores were estimated using a multivariable logistic regression model that included all baseline covariates listed in Table 2. Propensity score matching (PSM) was performed using 1:1 nearest-neighbor matching without replacement, with a caliper width of 0.2 standard deviations of the logit of the propensity score. The common support region was assessed by examining the distribution of propensity scores between groups. Patients without suitable matches within the specified caliper were excluded from the matched cohort. Covariate balance was assessed using standardized mean differences (SMDs), with a threshold of <0.1 indicating adequate balance. Absolute SMD plots were generated to visualize covariate balance before and after matching (Fig. S1-B, Supplemental Digital Content 4). To validate the primary outcome and adjust for baseline confounders, we applied inverse probability of treatment weighting (IPTW) and generated weighted cohorts using Pairwise Algorithmic (PA) weighting and Overlap Weighting (OW) methods. These approaches more truly reflect the independent association between aspirin and mortality. Finally, doubly robust estimation was employed to infer independent associations of the primary outcome between aspirin and non-aspirin groups. Subgroup analyses stratified by relevant covariates were conducted to mitigate potential survival bias. Sensitivity analysis using the E-value method evaluated the robustness of 28-day mortality results to unmeasured confounding.^[17]^

All analyses employed 2-tailed tests, with statistical significance defined as *P* < .05. Statistical procedures were executed using R (version 4.4.2; R Foundation for Statistical Computing, Vienna, Austria) and the FreeStatistics platform (version 2.1.1; Clinical Scientist Ltd, Beijing, China).

## 3. Results

### 3.1. Population selection process and characteristics of included populations

Figure 1 illustrates the detailed inclusion and exclusion procedures conducted in this study. A total of 9095 patients from the MIMIC IV database were identified as eligible, of whom 3207 patients (35.3%) were exposed to aspirin and 5888 (64.7%) were not. The mean age of the cohort was 66.6 years, and 5568 (61.2%) were male. The overall 28-day mortality rate was 25.5%, while that of the non-aspirin and aspirin groups was 30.0% (1767/5888) and 17.3% (556/3207), respectively. The baseline characteristics of patients are listed in Table 1.

**Figure 1.**
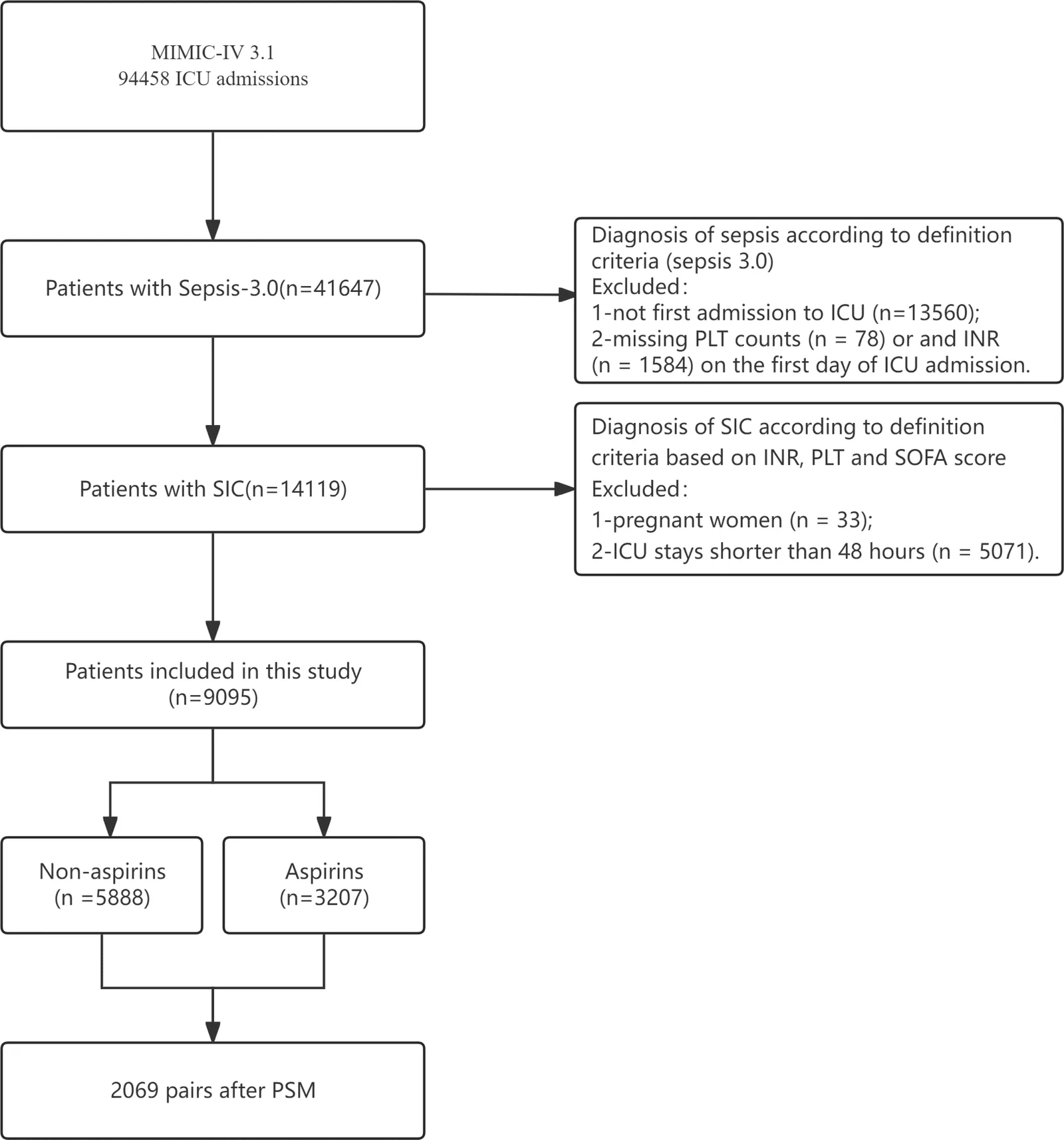
Flow chart of inclusion and exclusion criteria and grouping. ICU = intensive care unit, INR = international normalized ratio, MIMIC-IV = medical information mart for intensive care IV, PLT = platelet, PSM = propensity score matching, SIC = sepsis-induced coagulopathy, SOFA = sequential organ failure assessment.

After PSM, 2069 pairs of patients were successfully matched and subsequently included in the analysis. Figure S1-A, Supplemental Digital Content 4 displays the receiver operating characteristic curve assessing model discrimination efficacy, yielding an area under the curve of 82.2%, indicating good discriminative power in distinguishing aspirin groups. Post-matching standardized mean differences for all covariates were < 0.1, indicating adequate balance (Table 2 and Fig. S1-B, Supplemental Digital Content 4).

### 3.2. Association between aspirin exposure and primary outcome

Univariable Cox proportional regression demonstrated significantly reduced 28-day mortality with aspirin use (HR = 0.53, 95% CI: 0.49–0.59; *P* < .001). This association persisted after multivariable adjustment (adjusted HR, 0.70; 95% CI: 0.62–0.78; *P* < .001) and PSM (HR, 0.74; 95% CI: 0.65–0.85; *P* < .001), OW (HR, 0.72; 95% CI: 0.61–0.84; *P* < .001), PA (HR, 0.73; 95% CI: 0.61–0.84; *P* < .001), and IPTW (HR, 0.76; 95% CI: 0.7–0.84; *P* = .013). Doubly robust estimation further confirmed this association (HR, 0.76; 95% CI: 0.61–0.94; *P* < .001; Table 3). Consistently, Kaplan–Meier analysis revealed significantly lower 28-day mortality in aspirin versus non-users (Log-rank *P* < .0001; Fig. 2).

**Table 3:** Associations between aspirin use and 28-day mortality.

Analysis	28-d mortality(%)	*P*-value
No. of events/no. of patients at risk (%)		
Total	9095(25.5)	
Non-aspirins	1767/5888 (30.01)	
Aspirins	556/3207(17.34)	
Un-match crude analysis-hazard ratio (95% CI)	0.53 (0.49, 0.59)	<.001
Multivariable-hazard ratio (95% CI)*	0.7 (0.62, 0.78)	<.001
Adjust for propensity score (95% CI)†	0.72 (0.65, 0.81)	<.001
With propensity score matched (95% CI)‡	0.74 (0.65, 0.85)	<.001
Weighted.IPTW (95% CI)§	0.76 (0.7, 0.84)	.013
Weighted.PA (95% CI)‖	0.73 (0.64, 0.83)	<.001
Weighted.Ow (95% CI)¶	0.72 (0.61, 0.84)	<.001
Doubly robust with all cowariates#	0.76 (0.61, 0.94)	<.001

CI = confidence interval, IPTW = inverse probability of treatment weighting, HR = hazard ratio, Ow = overlap weighting, PA = propensity adjustment.

* Hazard ratio (HR) derived from a multivariable Cox proportional hazards model adjusted for all baseline covariates listed in Table 2.

† HR derived from a multivariable Cox proportional hazards model incorporating the same strata and covariates as in a, with additional adjustment for the propensity score.

‡ HR was derived from a multivariable Cox proportional hazards model applied to the propensity score-matched cohort (1:1 matching). The analysis included 4138 patients (2069 aspirin users and 2069 non-users).

§ HR was derived from a multivariable Cox proportional hazards model using inverse probability of treatment weighting (IPTW) based on the propensity score.

‖ HR was derived from a multivariable Cox proportional hazards model using propensity adjustment (PA) weighting.

¶ HR was derived from a multivariable Cox proportional hazards model using overlap weighting (Ow).

# HR was derived from a doubly robust estimator incorporating both propensity score weighting and direct adjustment for all covariates in Table 2.

**Figure 2.**
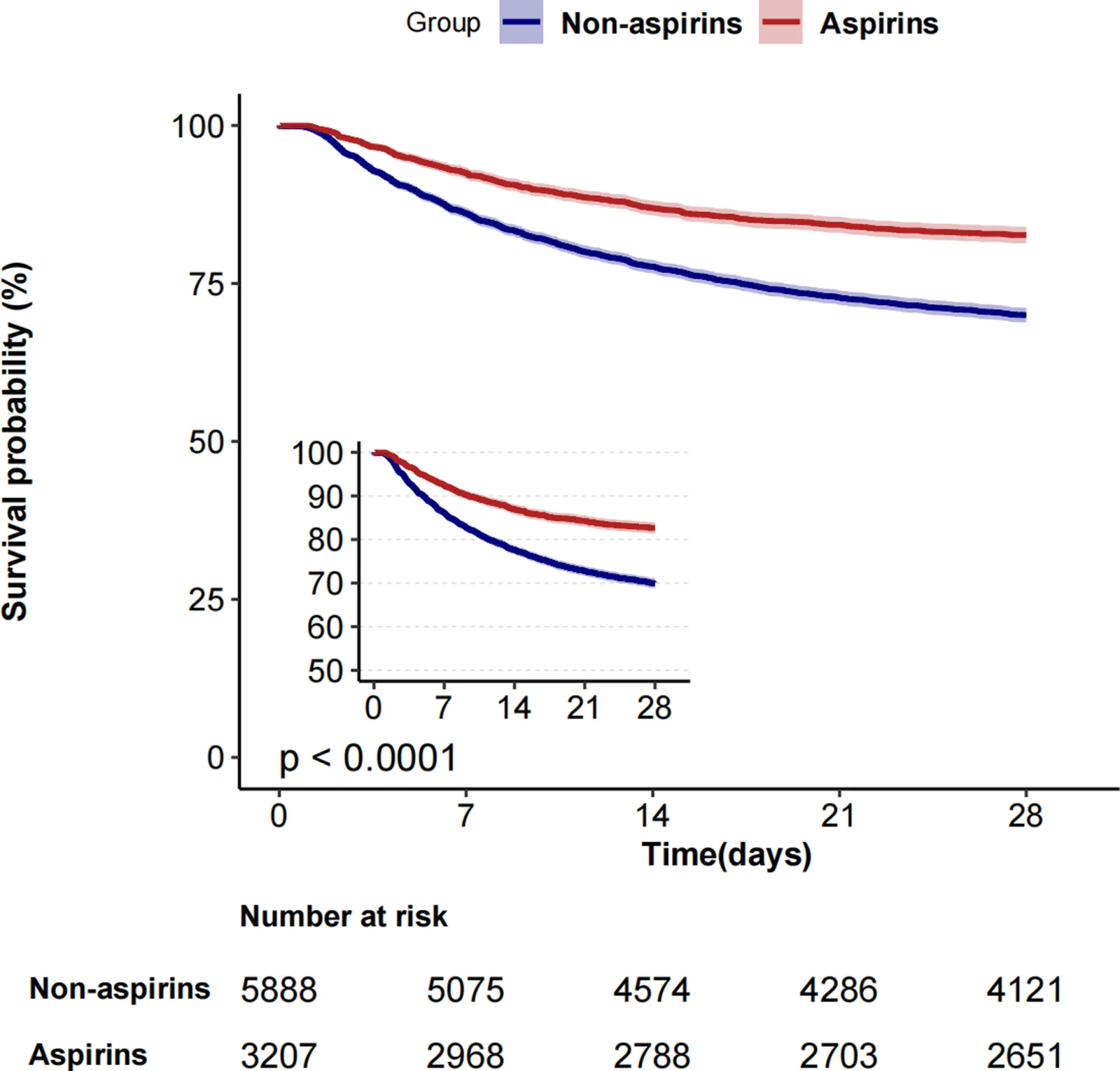
Kaplan–Meier survival curves of 28-day survival for sepsis-induced coagulopathy patients in the aspirin group and non-aspirin group.

### 3.3. Subgroup analyses

Subgroup analyses demonstrated a consistent protective association between aspirin use and reduced 28-day mortality across subgroups stratified by sex, race, age, body mass index, SOFA score, SIC score, malignancy, ICU heparin administration, continuous renal replacement therapy, and vasopressor use (Fig. 3). Significant treatment interactions (*P* interaction < .05) were observed for age (<65 vs ≥65 years), SIC score (<5 vs ≥5), and ICU heparin administration (Fig. 3).

**Figure 3.**
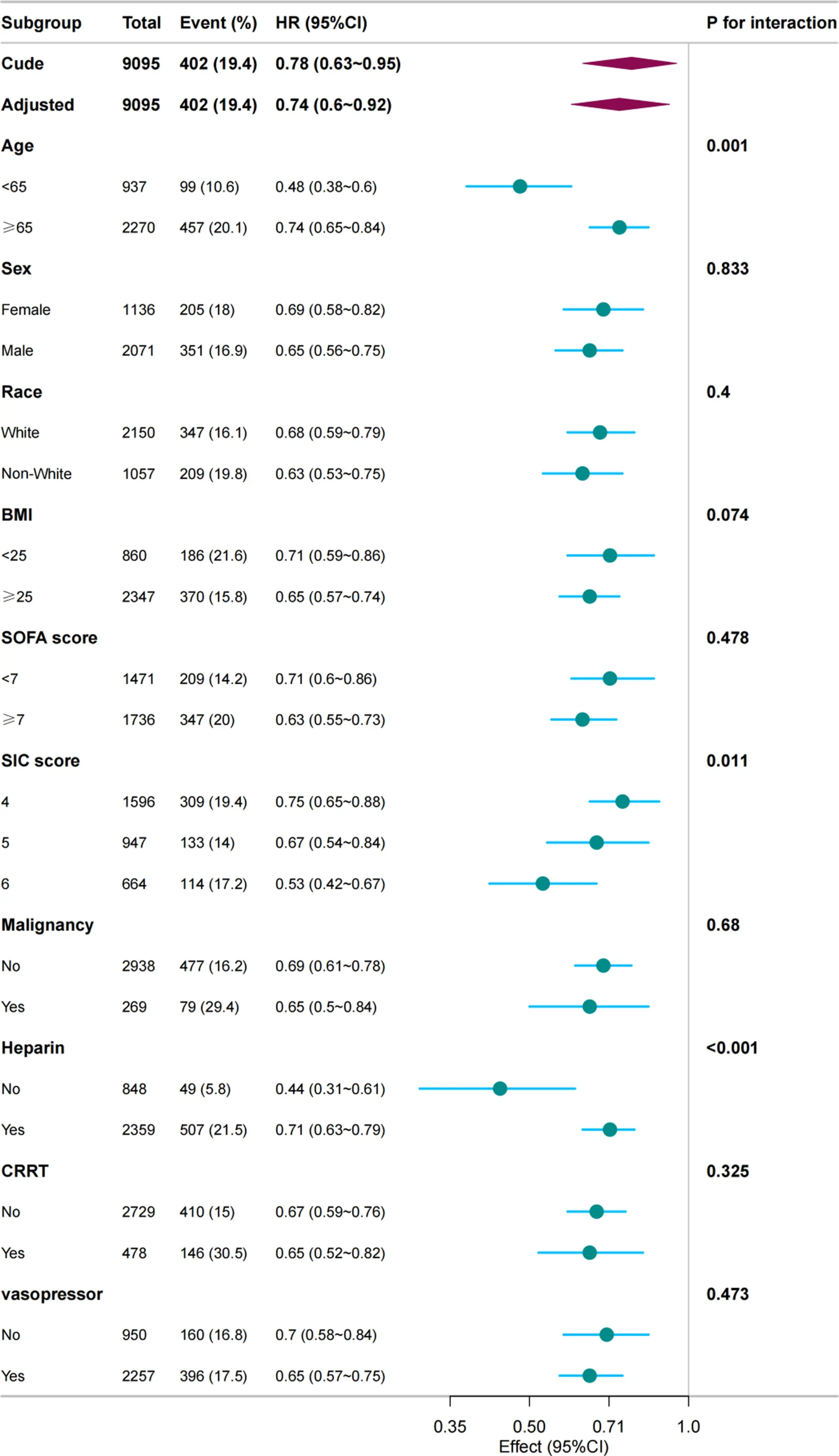
Subgroup analyses for the associations between Aspirin use and 28-day mortality in patients with sepsis-induced coagulopathy. Each stratification adjusted for all confounders (Table 2), except for the stratification factor itself. BMI = body mass index, CRRT = continuous renal replacement therapy, SOFA = sequential organ failure assessment.

### 3.4. Sensitivity analyses

Sensitivity analyses excluding patients with previous myocardial infarction, cerebrovascular disease, peripheral vascular disease, ICU heparin administration, pre-admission inpatient aspirin exposure, ICU stays < 48 hours, or missing covariates demonstrated similar associations (all *P* < .001; Table S4, Supplemental Digital Content 5). E-values (relative risk scale) for 28-day mortality were 1.77 (point estimate) and 1.48 (upper confidence limit; Fig. S2, Supplemental Digital Content 6).

### 3.5. Secondary outcomes

Inpatient aspirin exposure demonstrated statistically significantly reduced mortality (e.g., in-hospital, ICU, 60-day, 90-day, and 1-year) and increased organ support-free days (e.g., ICU, ventilator, and vasopressor) within 28 days (Table 4).

**Table 4:** Secondary outcome analyses.

Secondary outcomes	No. of events/patients (%)	Crude coefficient (95% CI)	Crude *P*-value	Adjusted coefficient (95% CI)*	Adjusted *P*-value
ICU-free days until 28 d		2.16 (1.67, 2.65)	<.001	0.81 (0.31, 1.3)	.001
MV-free days until 28 d		3.02 (2.51, 3.54)	<.001	1.4 (0.87, 1.92)	<.001
Vasopressor-free days until 28 d		2.65 (2.13, 3.17)	<.001	1.39 (0.86, 1.93)	<.001
In-hospital mortality	2033/9095 (22.35)	0.65 (0.59, 0.72)	<.001	0.77 (0.68, 0.87)	<.001
ICU mortality	1481/9095 (16.28)	0.56 (0.49, 0.63)	<.001	0.63 (0.55, 0.73)	<.001
60-d mortality	2787/9095 (30.64)	0.56 (0.51, 0.61)	<.001	0.71 (0.64, 0.78)	<.001
90-d mortality	3007/9095 (33.1)	0.57 (0.52, 0.62)	<.001	0.71 (0.65, 0.79)	<.001
1 yr mortality	3702/9095 (40.7)	0.6 (0.56, 0.65)	<.001	0.72 (0.66, 0.79)	<.001

CI = confidence interval, ICU = intensive care unit, MV = mechanical ventilation.

* Adjusted for all confounders (Table 2), except for the confounder itself.

## 4. Discussion

In this large retrospective cohort of critically ill patients with SIC, inpatient aspirin exposure was independently associated with significantly lower 28-day mortality, both before and after PSM adjustment. Subgroup and sensitivity analyses confirmed this protective association across diverse patient characteristics and clinical scenarios. This constitutes the first observational evidence evaluating aspirin exposure effects on 28-day mortality in SIC patients. Aspirin administration also correlated with improved secondary outcomes: reduced duration of mechanical ventilation, shorter vasopressor requirements, and decreased LOS-ICU. These findings suggest aspirin’s potential as an adjunctive immunomodulatory therapy in SIC management.

The pathogenesis of SIC involves a self-perpetuating cycle of inflammation and coagulation, potentially culminating in DIC and multiple organ dysfunction syndrome (MODS).^[2,3]^ Aspirin disrupts this pathogenic cycle in SIC through multiple mechanisms including inhibition of platelet activation, anti-inflammatory actions, and endothelial protection.^[18]^ First, by irreversibly inhibiting cyclooxygenase-1 (COX-1), aspirin attenuates thromboxane A2 production,^[19]^ mediating its antiplatelet effects. This suppresses platelet activation and aggregation, attenuating microthrombus formation. Second, aspirin decreases prostaglandin E2 levels by inhibiting COX-1, reducing vascular permeability and improving microcirculation. Aspirin downregulates pro-inflammatory cytokines (e.g., tumor necrosis factor-alpha [TNF-α], interleukin-1 beta [IL-1β]) via nuclear factor kappa B (NF-κB) blockade.^[18]^ Aspirin additionally promotes the resolution of neutrophil-mediated inflammation via the induction of 15-epi-lipoxinA4 and specialized pro-resolving mediators, such as aspirin-triggered resolvins, which limit inflammation and support tissue repair.^[20]^ Aspirin suppresses neutrophil extracellular trap (NET) formation (NETosis) by acetylating non-COX targets like high-mobility group box 1, thereby reducing thrombo-inflammatory organ damage.^[21,22]^ Furthermore, aspirin enhances nitric oxide (NO) bioavailability by acetylating endothelial nitric oxide synthase (eNOS) and inhibits NF-κB-mediated expression of adhesion molecules.^[23]^ These actions protect endothelial function, countering a hallmark pathological feature of SIC.

The therapeutic potential of aspirin in sepsis remains controversial, with conflicting evidence regarding its clinical efficacy. While some studies suggest potential benefits in specific patient subgroups, others report no significant effect on key outcomes. A study of 972 sepsis patients (including 267 with pre-admission antiplatelet therapy) found no significant association between prior antiplatelet use (predominantly aspirin) and sepsis severity, infection source, causative pathogens, development of organ failure/shock, or 90-day mortality.^[24]^ A large randomized controlled trial involving 16,703 community-dwelling adults aged ≥70 years demonstrated that prophylactic low-dose aspirin did not reduce sepsis-related mortality compared to placebo.^[25]^ Both of these findings provided indications of limited effectiveness. Recent retrospective research based on the large-scale MIMIC-IV database demonstrates evidence for potential benefit in subgroups. Aspirin use was reported to demonstrate significantly reduced mortality in patients with SA-MI,^[8]^ SA-AKI,^[7]^ SALI^[11]^ and SAE.^[9]^ Reduced mortality risk was observed in patients with SA-ARF receiving aspirin prior to ICU admission.^[10]^ These disparate findings suggest that the effect of aspirin in sepsis may be contingent upon specific patient factors, administration time, and clinical contexts. Notably, the pathophysiological milieu of SIC, characterized by more pronounced coagulation dysregulation and heightened thrombotic risk,^[2,3]^ may fundamentally alter aspirin’s pharmacological impact compared to sepsis without overt SIC.

Our findings are consistent with emerging evidence supporting anticoagulant and anti- inflammatory therapeutic strategies for SIC management. Peng et al^[26]^ demonstrated that UFH significantly reduced both 28-day and hospital mortality in SIC patients without elevating bleeding risk, affirming the prognostic benefit of anticoagulation in SIC. Similarly, Huang et al^[27]^ reported that dexmedetomidine, an agent possessing significant anti-inflammatory properties, reduced mortality and enhanced renal recovery in SIC patients. Furthermore, Yao et al^[28]^ observed that statin administration in the ICU significantly decreased in-hospital mortality among SIC patients. Together, these studies underscore the therapeutic value of mitigating inflammation and endothelial dysfunction in this critically ill population.

Building on these therapeutic concepts, the present study addresses a critical gap in the literature by investigating the specific clinical role of inpatient aspirin in SIC. To ensure the reliability of our findings, we employed robust statistical methodologies, including propensity score matching (PSM) and doubly robust estimation, which substantially mitigated confounding biases and strengthened our statistical inference. Our results suggest that inpatient aspirin exposure is independently associated with improved clinical outcomes in patients with SIC. However, due to the retrospective observational design, these findings must be interpreted with caution. They should be regarded as hypothesis-generating rather than definitive evidence supporting routine clinical use, and prospective randomized controlled trials (RCTs) are warranted before causal conclusions or formal therapeutic recommendations can be implemented.

It is worth noting that several previous studies have reported inconsistent findings; however, these discrepancies stem from fundamental differences in study design and patient populations. For instance, Tsai et al^[29]^ and Lavie et al^[30]^ observed no significant survival benefit associated with pre-admission or chronic antiplatelet/aspirin therapy in patients with sepsis. Similarly, the ANTISEPSIS^[25]^ randomized trial found that prophylactic low-dose aspirin did not reduce sepsis-related mortality among community-dwelling older adults. Unlike these prior studies – which evaluated chronic, pre-admission aspirin exposure or primary prevention cohorts – our analysis specifically targets acute, inpatient aspirin administration in critically ill patients with active, established SIC. This crucial distinction in both timing (acute inpatient intervention vs chronic pre-admission maintenance) and cohort selection (critically ill SIC patients vs general sepsis or healthy community-dwelling adults) likely explains the favorable survival association observed in our study.

Several limitations of this study should be acknowledged. First, despite the use of multiple propensity score-based methods (PSM, IPTW, OW, PA, doubly robust estimation) and E-value calculations, residual confounding by unmeasured variables remains possible. Factors such as frailty, do-not-resuscitate orders, specific infection sites, antibiotic regimens, and biomarkers (e.g., procalcitonin, C-reactive protein) were not available for adjustment. Confounding by indication is a particular concern, as patients who received aspirin may have differed from non-recipients in ways beyond measured covariates (e.g., perceived lower bleeding risk, better overall clinical condition). Observational studies cannot establish causality, and the possibility that the observed association reflects residual confounding rather than a true treatment effect cannot be excluded. Second, the absence of safety outcome data is a major limitation. Aspirin is associated with an increased risk of bleeding complications, but we were unable to reliably ascertain bleeding events from the MIMIC-IV database due to limitations in coding and documentation, as well as the inherent difficulty in distinguishing sepsis-related coagulopathic bleeding from aspirin-induced bleeding. This absence of safety data critically limits the clinical applicability of our findings. Third, although aspirin exposure was defined based on administration during ICU stay rather than survival to a treatment point, the potential for immortal time bias cannot be entirely excluded. Future studies employing time-dependent exposure modeling may further refine the causal inference. Fourth, information on aspirin dosing, duration, and treatment adherence was not available, precluding determination of optimal therapeutic regimens. Fifth, the study was derived from a single database (MIMIC-IV), which limits external validity; the results may not be generalizable to other healthcare settings or populations. Finally, the specific clinical indications for aspirin prescription were not directly available from the database, although the substantially higher prevalence of cardiovascular comorbidities among aspirin users suggests that most prescriptions were for secondary cardiovascular prevention. Prospective, randomized, double-blind trials are needed to confirm the efficacy and safety of specific aspirin dosing and timing regimens in SIC patients.

## 5. Conclusions

In conclusion, this retrospective cohort study found that inpatient aspirin exposure during ICU stay was independently associated with reduced 28-day mortality and improved secondary outcomes in critically ill patients with SIC across multiple analytical approaches. Given the observational design, potential residual confounding, immortal time bias, and unavailable bleeding safety data, these findings should be interpreted as an association rather than evidence of a causal therapeutic effect. Well-designed, multicenter randomized controlled trials are urgently needed to confirm the efficacy and safety of aspirin therapy in SIC patients before any clinical recommendations can be made.

## Acknowledgments

The authors thanks Dr Qilin Yang for guidance on data extraction.

## Author contributions

**Conceptualization:** Jiantao Zheng, Yinying Wang, Siming Lin.

**Data curation:** Jiantao Zheng.

**Formal analysis:** Jiantao Zheng, Chanjuan Zhou.

**Funding acquisition:** Siming Lin.

**Methodology:** Jiantao Zheng, Yinying Wang, Siming Lin.

**Supervision:** Yinying Wang.

**Visualization:** Chanjuan Zhou.

**Writing – original draft:** Jiantao Zheng, Yinying Wang.

**Writing – review & editing:** Jiantao Zheng, Yinying Wang, Chanjuan Zhou, Siming Lin.












