# Supplementary material for: Association between inpatient aspirin exposure and 28-day mortality in critically ill patients with sepsis-induced coagulopathy: A cohort study
Source: Medicine (Baltimore). 2026 Nov 6;105(45):e51494. doi: 10.1097/MD.0000000000051494 (PMC13747455; doi:10.1097/MD.0000000000051494)
Supplement: Supplementary file 1 [file medi-105-e51494-s001.doc]

**Table S1 Diagnosis criteria of sepsis-induced coagulopathy**

|  | **points** | **items** |
| --- | --- | --- |
| PLT count (10^9^/L) | 2 | < 100 |
|  | 1 | ≥ 100, <150 |
| INR | 2 | > 1.4 |
|  | 1 | >1.2, ≤1.4 |
| Total SOFA score | 2 | ≥ 2 |

Diagnostic threshold: A total score ≥4 points confirms sepsis-induced coagulopathy.

INR, international normalized ratio; PLT, platelet; SOFA, Sequential Organ Failure Assessment.
