# Supplementary material for: Association between inpatient aspirin exposure and 28-day mortality in critically ill patients with sepsis-induced coagulopathy: A cohort study
Source: Medicine (Baltimore). 2026 Nov 6;105(45):e51494. doi: 10.1097/MD.0000000000051494 (PMC13747455; doi:10.1097/MD.0000000000051494)
Supplement: Supplementary file 2 [file medi-105-e51494-s002.doc]

**Table S2 Univariate Cox regression analysis of the association between risk factors and 28-day mortality**

| **Variables** | **HR(95%CI)** | ***P*** |
| --- | --- | --- |
| Age (years) | 1.01 (1.01,1.02) | < 0.001 |
| Gender: Female vs Male | 0.87 (0.8,0.95) | < 0.001 |
| Race: non-white vs white | 1.33 (1.22,1.44) | < 0.001 |
| BMI (kg/m²) | 0.9948 (0.9878,1.0019) | 0.15 |
| Heart rate (beats/min) | 1.01 (1.01,1.01) | < 0.001 |
| Mean blood pressure (mmHg) | 0.9908 (0.9864,0.9953) | < 0.001 |
| RR (breaths/min) | 1.06 (1.06,1.07) | < 0.001 |
| Body temperature (°C) | 0.69 (0.65,0.73) | < 0.001 |
| SpO_2_ (%) | 0.91 (0.9,0.93) | < 0.001 |
| Glucose (mg/dL) | 1 (1,1) | 0.501 |
| Hemoglobin (g/dL) | 0.99 (0.97,1.01) | 0.26 |
| PLT (×10^9^/L) | 1.0003 (0.9999,1.0007) | 0.129 |
| WBC (×10^12^/L) | 1.0031 (1.0014,1.0048) | < 0.001 |
| AG (mmol/L) | 1.06 (1.06,1.07) | < 0.001 |
| Bicarbonate(mmol/L) | 0.95 (0.94,0.96) | < 0.001 |
| BUN (mg/dL) | 1.01 (1.01,1.01) | < 0.001 |
| Calcium(mg/dL) | 1.01 (0.97,1.06) | 0.555 |
| Creatinine (mg/dL) | 1.09 (1.07,1.11) | < 0.001 |
| Sodium (mmol/L) | 0.9903 (0.9834,0.9972) | 0.006 |
| Potassium(mmol/L) | 1.12 (1.08,1.15) | < 0.001 |
| PTT (seconds) | 1.0053 (1.0043,1.0064) | < 0.001 |
| PT(seconds) | 1.01 (1.01,1.01) | < 0.001 |
| INR | 1.1 (1.09,1.12) | < 0.001 |
| Lactate (mmol/L) | 1.04 (1.04,1.05) | < 0.001 |
| MI: yes vs no | 1.11 (1,1.23) | 0.043 |
| CHF: yes vs no | 1.16 (1.07,1.26) | < 0.001 |
| PVD: yes vs no | 0.87 (0.78,0.98) | 0.027 |
| CVD: yes vs no | 1.36 (1.22,1.52) | < 0.001 |
| COPD: yes vs no | 1.1 (1,1.2) | 0.054 |
| Rheumatic disease: yes vs no | 1.1 (0.89,1.35) | 0.371 |
| Peptic ulcer disease: yes vs no | 1.12 (0.92,1.37) | 0.255 |
| Diabetes: yes vs no | 0.95 (0.87,1.04) | 0.302 |
| CLD: yes vs no | 1.7 (1.57,1.86) | < 0.001 |
| Malignancy: yes vs no | 2.01 (1.83,2.21) | < 0.001 |
| AKI: yes vs no | 3.23 (2.57,4.05) | < 0.001 |
| CCI | 1.15 (1.13,1.16) | < 0.001 |
| APS III score | 1.02 (1.02,1.03) | < 0.001 |
| SAPS II score | 1.04 (1.03,1.04) | < 0.001 |
| OASIS score | 1.06 (1.05,1.06) | < 0.001 |
| SOFA score | 1.12 (1.11,1.13) | < 0.001 |
| SIC score (ref=4) |  |  |
| 5 | 0.91 (0.82,1.01) | 0.088 |
| 6 | 1.45 (1.32,1.59) | < 0.001 |
| Heparin: yes vs no | 1.43 (1.28,1.59) | < 0.001 |
| vasopressor: yes vs no | 1.22 (1.12,1.33) | < 0.001 |
| MV: yes vs no | 1.29 (1.18,1.41) | < 0.001 |
| CRRT: yes vs no | 2.08 (1.9,2.29) | < 0.001 |
| Los.ICU | 0.9916 (0.986,0.9973) | 0.004 |

AG, anion gap; AKI, Acute Kidney Injury; APS III, Acute Physiology Score III; BMI, body mass index; BUN, blood urea nitrogen;CCI, Charlson comorbidity index; CHF, congestive heart failure; CLD, chronic liver disease; COPD, chronic obstructive pulmonary disease; CRRT, continuous renal replacement therapy; CVD, cerebrovascular disease; ICU, intensive care unit; INR, international normalized ratio; MI, myocardial infarction; MV, Mechanical ventilation; OASIS, Oxford acute severity of illness score;PT, prothrombin time; PTT, partial thromboplastin time; PVD, peripheral vascular disease; PLT, platelets; RR, respiratory rate; SAPS II, simplified acute physiology score II; SIC, sepsis-induced coagulopathy; SOFA, sequential organ failure assessment; SpO₂, oxygen saturation; WBC, white blood cell count.
