# Supplementary material for: Association between inpatient aspirin exposure and 28-day mortality in critically ill patients with sepsis-induced coagulopathy: A cohort study
Source: Medicine (Baltimore). 2026 Nov 6;105(45):e51494. doi: 10.1097/MD.0000000000051494 (PMC13747455; doi:10.1097/MD.0000000000051494)
Supplement: Supplementary file 3 [file medi-105-e51494-s003.doc]

**Table S3 Missing number (%) for included variables in the datasets**

| **Variable** | **Missing Count** | **Missing Percent(%)** |
| --- | --- | --- |
| AG | 19 | 0.2089 |
| BMI | 2458 | 27.0258 |
| BUN | 3 | 0.033 |
| Lactate | 2104 | 23.1336 |
| RR | 4 | 0.044 |

AG, anion gap; BMI, body mass index; BUN, blood urea nitrogen; RR, respiratory rate.
