# Supplementary material for: Association between inpatient aspirin exposure and 28-day mortality in critically ill patients with sepsis-induced coagulopathy: A cohort study
Source: Medicine (Baltimore). 2026 Nov 6;105(45):e51494. doi: 10.1097/MD.0000000000051494 (PMC13747455; doi:10.1097/MD.0000000000051494)
Supplement: Supplementary file 4 [file medi-105-e51494-s005.doc]

**Table S4 Sensitivity analyses**

| **Variable** | **No. of patients** | **No. of events(%)** | **Crude HR (95%CI)** | **Crude P-value** | **Adjusted HR (95%CI)^a^** | **Adjusted p-value** |
| --- | --- | --- | --- | --- | --- | --- |
| Non-MI , Non-CVD and Non-PVD | 5566 | 1360(24.43) | 0.41 (0.35,0.48) | <0.001 | 0.60 (0.50,0.71) | <0.001 |
| Non-heparin exposure | 2101 | 411(19.56) | 0.17 (0.13,0.24) | <0.001 | 0.42 (0.3,0.59) | <0.001 |
| Non aspirin exposure pre-ICU | 7728 | 2143(27.73) | 0.6 (0.54,0.67) | <0.001 | 0.7 (0.62,0.79) | <0.001 |
| Include patients stayed in lCU< 48 hours | 14152 | 3426(24.20) | 0.44 (0.41,0.48) | <0.001 | 0.62 (0.55,0.71) | <0.001 |
| Exclude patients with missing covariates | 5482 | 2324(23.75) | 0.41 (0.36,0.47) | <0.001 | 0.64 (0.55,0.74) | <0.001 |

^a^Adjusted for confounders (Table 2).

CI: confidence interval; MI: myocardial infarction; CVD, cerebrovascular disease; ICU: intensive care unit.
